# Editorial: Mapping the unseen: advancements and innovations in spatial epidemiology for disease dynamics and public health interventions

**DOI:** 10.3389/fepid.2026.1934467

**Published:** 2026-07-31

**Authors:** Wei Wang, Pengpeng Ye

**Affiliations:** 1 Center for Clinical and Epidemiologic Research, Beijing Anzhen Hospital, Capital Medical University, Beijing, China; 2 Beijing Institute of Heart, Lung and Blood Vessel Diseases, Chinese Center for Disease Control and Prevention, Beijing, China; 3 National Center for Chronic and Noncommunicable Disease Control and Prevention, Chinese Center for Disease Control and Prevention, Beijing, China

**Keywords:** disease mapping, geographic information systems (GIS), health inequalities, public health interventions, spatial epidemiology

In the wake of the COVID-19 pandemic, the demand for geographically precise disease intelligence has never been clearer. Spatial epidemiology—at the intersection of geography, epidemiology, and statistics—has moved beyond simply mapping disease occurrence (1). It now explains why risks cluster where they do, and how place shapes health over time. Advances in GIS, remote sensing, and computational modeling allow researchers to detect hotspots and inform targeted interventions with unprecedented granularity (2). We assembled this research topic to compile innovations that bridge analytic methods with public health action.

The thirteen contributions gathered here span Africa, Asia, and the Americas, and range from classic disease mapping to real-time digital surveillance. These studies illustrate that spatial epidemiology is no longer confined to describing where disease occurs; it increasingly explains why geographic inequalities persist, how patterns evolve, and what interventions are feasible in specific local contexts. During the editorial process, we were struck by the geographic and methodological diversity of the submissions, which collectively push the field toward greater integration, precision, and practical relevance. We also noticed a recurring constraint: routine health information systems remain too weak to support the sophisticated models being proposed (3). This tension runs through the Research Topic. It shapes what spatial epidemiology can currently deliver, and where it must improve.

Several papers advance disease mapping through spatially adaptive modeling. In a nationwide analysis of China, Hu et al. reveal a pronounced east-to-west gradient in life expectancy and show that the health effects of SO_2_, NO_2_, and PM_10_ vary markedly by region—underscoring the limits of uniform national air-quality standards. Similarly, Gebrehana et al. apply multiscale geographically weighted regression to anemia among women of reproductive age in Mozambique, identifying place-varying predictors including water source, pregnancy status, and nutritional factors that national surveys typically smooth over. These studies remind us that averaging across space can obscure the very inequalities that public health programs most need to address, and that locally tailored modeling is essential for equitable resource allocation. Habtewold and Arero extend this approach to child undernutrition in Ethiopia, comparing Bayesian spatial models—including conditional autoregressive structures—to identify high-risk regions and modifiable determinants ranging from maternal education and breastfeeding practices to household sanitation and wealth. Viewed alongside the Mozambique findings, the message is consistent: fine-scale spatial targeting outperforms national averages when resources are scarce and needs are heterogeneous, offering a clear rationale for geographically nuanced nutrition programs. Yet they also share a limitation. Cross-sectional data could capture spatial pattern well, but struggle to establish temporal causality.

A second theme concerns spatial accessibility and its distorting effect on disease surveillance. In rural Kenya, Musau et al. show that longer travel time to emergency care correlates with more severe pediatric anemia at presentation—not merely delaying care, but selectively filtering which children reach facilities. Chen et al. observe a parallel pattern in Taiwan: greater distance to occupational medicine clinics is associated with systematic underreporting of milder cases. Both studies caution that facility-based disease maps reflect not only true epidemiological burden but also systemic barriers to diagnosis and reporting, a bias that spatial analysts must actively account for when interpreting geographic patterns. Distance acts as an unmeasured confounder that can mislead both clinicians and policymakers if ignored.

Infectious disease surveillance and vaccination monitoring are also prominent in this Research Topic. Muse et al. map measles cases and vaccination coverage in eastern Ethiopia's Somali region, pinpointing areas where high burden and low coverage overlap—precisely the zones where reactive vaccination campaigns are most urgently needed. Yet Moturi et al., comparing routine and survey-based estimates in Western Kenya, expose substantial discrepancies between data sources. This tension is instructive: even the most sophisticated spatial model is only as reliable as the denominator beneath it. Data quality, surveillance infrastructure, and health system governance, not merely analytic technique, ultimately determine what maps can truthfully show. For practitioners, this means that strengthening routine information systems may do more for epidemic preparedness than adopting novel algorithms alone.

Turning to epidemic dynamics, Nadarajah and Umar apply extreme value analysis to COVID-19 across 54 African countries, modeling monthly maxima to anticipate surges that could overwhelm fragile health systems. Vasconcelos et al. examine COVID-19 patterns in the eastern Amazonian transition zones, where hospitalization and mortality rates reveal sharp territorial boundaries tied to socioeconomic inequality and environmental vulnerability. Both studies highlight that epidemic preparedness requires more than average-case planning; it demands spatially explicit contingency strategies capable of responding to extreme events in the most vulnerable locations. Such approaches are particularly vital in regions where geographic inequality and limited health system capacity compound each other.

Environmental and climate risks receive careful attention as well. Tesfa et al. track rising chronic respiratory disease morbidity in Ethiopia's charcoal-producing regions, identifying seasonal peaks that should guide proactive resource allocation. Murray and Ignaszak step back to examine how climate change amplifies epidemic risk through multiple environmental pathways. Despite differing scales—one local and time-series, the other global and conceptual—both contributions argue for surveillance systems that integrate environmental monitoring with health data, a need that will only intensify as climate volatility increases and vulnerable populations face compounding exposures.

The Research Topic also stretches spatial epidemiology into the life course and digital realms. Xia et al. link birth residence in Massachusetts to later addiction service utilization, using generalized additive models with spatial smoothing to show that early neighborhood disadvantage leaves lasting geographic marks across decades. Ma and Zhao propose a policy-aware transformer for video-based surveillance—an ambitious integration of real-time health monitoring and machine learning that, while promising, forces us to confront questions of privacy, equity, and public trust that technology alone cannot resolve. These contributions illustrate that spatial epidemiology now reaches from birth certificates to video feeds, expanding its scope while raising new ethical responsibilities.

Methodologically, this topic traces a clear trajectory: from spatially adaptive models that detect local hotspots, through Bayesian and geostatistical approaches that quantify uncertainty, to machine learning systems that process real-time streams. Temporal integration—forecasting, extreme value modeling, and seasonal decomposition—runs throughout these contributions, reflecting a field that increasingly thinks not just in static maps, but in dynamic timelines and predictive horizons that anticipate health needs before they become crises.

Critically, these studies do not stop at description. They demonstrate how spatial evidence can guide vaccine deployment, emergency care routing, and environmental control—yet they also warn that translating maps into action demands careful attention to local context, data quality, community relevance, and health system feasibility. Spatial epidemiology is most powerful when it serves as a bridge between analytic insight and policy deliberation, rather than as an academic exercise detached from implementation realities. We should be honest about this. The field has produced elegant maps and sophisticated models. Without closing the implementation gap—getting those maps to decision-makers and proving they improve health outcomes—it will not achieve greater success (4).

As guest editors, we see this Research Topic as evidence that spatial epidemiology has entered a more integrative and action-oriented phase. The field is no longer satisfied with asking where disease occurs; it increasingly demands to know why inequalities persist, how patterns evolve, and what specific actions are feasible in specific places. Achieving this will require deeper integration of remote sensing, mobility data, routine health information systems, and community-based knowledge—not as technical add-ons, but as core components of equitable surveillance (5). We hope this topic fosters the interdisciplinary collaboration necessary to realize that vision. By mapping the unseen, these studies help transform geographic variation into public health intelligence that is not only precise, but locally relevant and ethically grounded. Ultimately, understanding place is fundamental to understanding disease dynamics, and spatially informed evidence remains indispensable for building public health strategies that are as geographically precise as the problems they aim to solve.
